# Occupational Complexity and Late-Life Global Cognition: The SONIC Study

**DOI:** 10.1093/geroni/igaa057.2823

**Published:** 2020-12-16

**Authors:** Yoshiko Ishioka, Yasuyuki Gondo, Yukie Masui, Takeshi Nakagawa, Hiroki Inagaki, Madoka Ogawa, Saori Yasumoto, Tatsuro Ishizaki

**Affiliations:** 1 Keio University, Minato-ku, Kanagawa, Japan; 2 Osaka University, Suita, Osaka, Japan; 3 Tokyo Metropolitan Institute of Gerontology, Itabashi-ku, Tokyo, Japan; 4 National Center for Geriatrics and Gerontology, Ōbu, Aichi, Japan

## Abstract

Studies have reported that work exposure to cognitively demanding environments predicted the level of late-life cognitive abilities. To date, whether or not such a relationship between work complexity and cognitive function is maintained in very old adults remains unknown. In the present study, we examined how the associations between lifetime work’s complexity and global cognition vary by age groups (70s, 80s, and 90s). To this end, we used data from 2754 Japanese community-dwelling participants in the SONIC Project. Specifically, we tested multiple group path models comparing the models based on differences in age and gender. The effects of work complexities on global cognition were found for male septuagenarians and octogenarians, having controlled for the variables related to education. The relationships between them were marginally significant for male nonagenarians. Based on the analysis, we discuss the maintenance of cognitive reserve and implications for cognitive and physical health in very old ages.

